# Editorial: Evaluation of quality and safety of agricultural products by non-destructive sensing technology

**DOI:** 10.3389/fpls.2023.1203029

**Published:** 2023-05-26

**Authors:** Jiangbo Li, Yuzhen Lu, Jianwei Qin

**Affiliations:** ^1^ Intelligent Equipment Research Center, Beijing Academy of Agriculture and Forestry Sciences, Beijing, China; ^2^ Department of Biosystems & Agricultural Engineering, Michigan State University, East Lansing, MI, United States; ^3^ Environmental Microbial and Food Safety Laboratory, Agricultural Research Service, U.S. Department of Agriculture, Beltsville, MD, United States

**Keywords:** agricultural products, crop phenotype, non-destructive sensing techniques, quality assessment and control, smart agriculture-food systems

Quality assessment is an essential task in post-harvest processing of agricultural products and important for improving their economic value. Advanced non-destructive sensing technologies, in conjunction with data analytics and control and automation technology, have evolved as a potent means for augmenting food quality control efforts. This research topic covers the latest applications of various sensing technologies, including machine vision, near-infrared spectroscopy, hyperspectral imaging, spatial-frequency domain imaging and ultrasound technology, in the quality assessment of fruits, vegetables, edible oils and seeds as well as the analysis of crop phenotypes.

Fruits are important agricultural products. Rapid, non-destructive, and accurate quality evaluation and grading can add value to the commodities when delivered to the marketplace. The soluble solids content (SSC) is one of the key internal quality characteristics of fruits. Yang et al. used the visible and near-infrared spectroscopy to assess SSC in Korla fragrant pears. A combination of bootstrapping soft shrinkage (BOSS) and successive projections algorithm (SPA) was used to extract important wavelengths from full-spectrum data. The partial least squares (PLS), least squares support vector machine (LS-SVM) and multiple linear regression (MLR) models were built for SSC predictions. The study showed that the PLS model based on 17 wavelengths selected by BOSS-SPA obtained the best prediction with *r*_p_ of 0.94 and RMSEP (root mean square error of prediction) of 0.27%. In fruit quality prediction, the model robustness is crucial for practical implementation. Traditional near-infrared spectroscopy that performs aggregated measurements is limited in decoupling absorption and scattering effects of biological tissues. Spatial-frequency domain imaging (SFDI) has emerged as a means for quantifying and mapping tissues optical properties, which can be useful for fruit quality assessment. By demodulating the reflectance images under structured illuminations with changed frequencies and phases, the absorption coefficient and reduced scattering coefficients of biological samples can be estimated by an inversion algorithm based on appropriate light transfer models. Peng et al. reported on the measurement of optical properties of apples at the wavelengths of 460, 527, 630 and 710 nm using the SFDI technique, for assessing the SSC, firmness, and color parameters. The resultant absorption coefficient and reduced scattering coefficients were utilized for building models of SVM, MLR and PLS to predict apple quality attributes. The monitoring of quality changes during postharvest is important to quality control. Fruit usually undergoes certain periods of storage and transportation after harvest and before sale, in which they may be susceptible to spoilage and quality deterioration. The rapid, *in situ* identification of fruit spoilage is beneficial for minimizing product and financial losses. The volatile compounds (e.g., alcohols, esters, terpenes, and ethylene) released from fruit reflect fruit quality status during storage. The qualitative and quantitative analyses of these volatile compounds provide valuable insights into fruit quality. Zhou et al. used a spiral silver halide fiber optic evanescent wave spectroscopy (FOEW) sensor to explore the feasibility of identifying volatile compounds released from grapes *in situ*. The absorption peaks of ethanol in the volatile compounds were found in the FOEW spectra and their intensity gradually increased as the storage time of the grapes increased. Principal component analysis (PCA) of the spectra showed the clustering at different storage times, revealing that the concentration of the ethanol released from the grapes changed significantly with time. They built PLS discriminant analysis model for classifying grape samples as “fresh”, “slight spoilage” or “severe spoilage”, achieving the validation accuracy of 100%. This study can provide a reference for rapid identification of fruit deterioration.

In vegetable quality testing, cabbage is one of the economically important vegetable products worldwide. The dents and cracks of cabbage caused by extrusion and collection during transportation negatively impact both the commercial value and storage time of the commodity. Consumer-grade RGB-D (red-green-blue-depth) cameras are being increasingly used in the agriculture and food domain, which integrate the functionality of color (RGB) and depth (or range) sensing to provide richer information, and particularly the 3D point cloud from depth channel data enables the reconstruction of object surface geometry and shape, which can be useful for quality assessment of agricultural produce. Curvature is an important feature in shape analysis and can be used for the detection of shape defects. Gu et al. used an Intel RealSense-D455 depth camera to obtain the 3D point cloud cabbage segmented from the background noise through preprocessing and region of interest extraction. The normal vector was estimated based on a least-squares plane fitting method, and the curvature threshold was defined in agreement with the curvature character parameters. The surface defect detection was realized according to the curvature difference between the normal area and the defective area on the cabbage surface.

Seed quality is crucial for the productivity and eventual products quality of crops. Aged seeds generally have low plant vigor and growth, which need to be identified and segregated out. Wang et al. employed short wave-near infrared hyperspectral reflectance imaging to identify aged maize seeds. ANOVA was used to reduce data dimensionality. The band ratio (1987 nm/1079 nm) selected by ANOVA from embryo-side spectra achieved 95% classification accuracy. The image texture features, including histogram statistics and gray-level co-occurrence matrix, were extracted from the band ratio image to establish fusion models, yielding the accuracy of 97.5%. This study indicated that imaging at two wavelengths combined with the extraction and modeling of image textural features could detect aged maize seeds effectively. Cracks of cottonseeds negatively influence the germination rate of the crop. It can be a challenging task for techniques such as machine vision, spectroscopy, and thermal imaging, to detect slight cracks in the cottonseeds. An acoustic method is potentially sensitive to localizing fine structure defects. Zhang et al. presented a novel methodology to detect slightly cracked cottonseed using air-coupled ultrasound with a lightweight vision transformer and a sound-to-image encoding method. The echo signal of air-coupled ultrasound from cottonseeds was obtained in a non-contact way. The intrinsic mode functions of the ultrasound signal were obtained as the sound features through variational mode decomposition, which were further converted into color images by a color encoding method. A lightweight MobileViT model was trained with the resultant color image to discriminate between the slightly cracked and normal cottonseeds, resultant in an overall recognition accuracy of 90.7%.

In addition to quality assessment of raw or fresh-market commodities, the non-destructive sensing technology can also be applied to processed products. Pomegranate kernel oil has gained global attention due to the health benefits associated with its consumption, especially fatty acid composition. Various analytical methods have been used for quality control of edible fats and oils. However, these methods are expensive, labor-intensive, and often require specialized sample preparation, restricting their applicability on a commercial scale. Okere et al. used Fourier transform near-infrared and mid-infrared spectroscopy to qualitatively and quantitatively predict quality attributes of the pomegranate kernel oil. PCA and orthogonal partial least squares discriminant analysis were applied for qualitative analysis, and PLS regression was used for developing quantitative models. Their results demonstrated the potential of near- and mid-infrared spectroscopy for rapid screening of pomegranate oil quality.

In addition to aforementioned studies, this research topic also includes two articles pertaining to crop phenotypic analysis. Watermelon is a widely consumed and nutritious fruit with rich sugars. Abiotic stresses caused by changes in temperature, moisture, etc., pose a threat to crop production and product quality. Stress diagnosis based on monitoring plant morphological features (e.g., shape, color, and texture) is important for optimizing management practices for yield and quality protection. Nabwire et al. classified watermelon plants exposed to low-temperature stress conditions from the normal ones using features extracted by image analysis, which achieved 100% accuracy in discriminating between normal and low-temperature stressed watermelon plants. They also built models for estimating the number of leaves and plant age using the extracted features, resulting in *R*_2_, RMSE and mean absolute difference (MAD) of 0.94, 0.87 and 0.88, respectively for the number of leaves and the *R*_2_ and RMSE of 0.92 and 0.29 (weeks), respectively, for estimating the plant age. The models developed can be utilized for monitoring and analysis of phenotypic traits during watermelon growth. The segregation of plug seedlings by quality is important for planting high-quality seedlings for wide-scale production. Du et al. used a color vision system to acquire images of the tops of pepper plug seedlings and built an EfficientNet based convolutional neural network (CNN) model for classifying the plug seedlings into three classes (strong seedlings, week seedlings, and empty plug cells). The CCN model, structured by adding a convolutional block attention module (CBAM) to EfficientNet-B7, alongside transfer learning and Adam optimization, achieved an accuracy of about 98%. The proposed method had high accuracy for the plug seedling quality classification task.

Non-destructive sensing technologies reported in the above studies provide useful tools for the assessment of agricultural product quality and analysis of crop phenotypes. These studies provide valuable references for further research and developments of advanced sensing technologies for enhanced quality assessment and control. Based on these technologies, the advanced detection and monitoring equipment for real-time, high-volume application has been and is being developed to propel the production of smart agriculture-food systems.

## Author contributions

All authors have made substantial contributions to the work reported in the Editorial. Specific contributions are as follows: JL: Original Editorial writing. YL: Editing. JQ: Editing. All authors contributed to the article and approved the submitted version.

